# The Story of the Insane from Year to Year

**Published:** 1899-01-14

**Authors:** 


### THE STORY OF THE INSANE FROM YEAR TO YEAR.

(ELEVENTH SERIES.)

### STAFFORDSHIRE COUNTY ASYLUM AT LICHFIELD.

THIS institution contained 763 patients on January 1st, 1897, and by the beginning of 1898 the number had risen to 782. There were 227 admissions, 27 readmissions, 92 discharged recovered, 14 discharged relieved, and 120 deaths. Put into percentages, we find that the recoveries stand at 40 calculated on the admissions. This is about the usual average in county asylums, and is somewhat higher than their own average for the last 23 years, which stands at 34·12. The deaths, calculated on the average number resident, are given at 15·38 per cent., which is also above their average, namely, 11·96. Turning to Table X. we notice that in the year's admissions 24 of the patients owed their insanity to various moral causes, 36 to drink, 30 to hereditary tendency, and 40 to previous attacks, while in 77 instances the cause was not ascertained. It is an undoubted fact that there has been of late a tendency to transfer insane patients from workhouses to asylums who in former years would have been allowed to end their days in the workhouse. This course cannot be altogether regretted, as the poor old lunatic passes his last months in comparative comfort; but it has had the effect of increasing the medical and nursing work of asylums; so much so at the Lichfield Asylum that Dr. Spence says it is becoming a hospital in fact if not in name. The asylum is being considerably added to and altered. The weekly cost of maintenance is 8s. 8½d. per head.

### DERBY BOROUGH ASYLUM AT ROWDITCH.

HERE the numbers rose slightly during the year, being 305 on January 1st, 1897, and 318 on the last day of December. There were 60 first admissions, 22 readmissions, 28 recoveries, eight discharged relieved, and 33 deaths. The recoveries were 36·8 per cent. on the average number resident. This is a fair proportion, but is considerably under their own average, which, since the opening of the asylum in 1888, stands at 46·4. The deaths were 10·3 per cent. on the average number resident. Of these deaths 22 were due to cerebral and spinal diseases, one to pleurisy, and two to peritonitis, while consumption does not appear at all among the death returns, and this is about the only time we have missed it in the tables of our public asylums. Of the year's admissions 19 are stated to owe their insanity to various moral causes, domestic trouble being the most frequent of these; 20 to intemperance in drink, 23 to hereditary tendency, and the same number to previous attacks. The asylum is practically full, and Dr. Macphail's attempt to reduce the numbers by sending apparently suitable cases to the workhouse has failed, as it has so often done elsewhere, for he tells us that each case sent to the workhouse has been returned. The average age at death of the 33 patients who died in 1897 was a little over 48 years, and Dr. Macphail points out that the great risk of death in insanity "is not after it has lasted several years and become chronic, but in the first and second years." We are informed that of the staff four nurses and attendants have obtained the certificate in mental nursing. It is not stated whether this is the certificate of the Medico-Psychological Association or that of the Derby Borough. We hope it is the latter, as Dr. Macphail is unquestionably one of the ablest of our medical superintendents, and we earnestly hope he is not ashamed to put his own stamp on his own article. The weekly charge is 9s. 11d. per head.

### DISTRICT ASYLUM, CORK.

ON January 1st, 1897, there were in residence 1,358 patients, and at the end of the year this number had risen to 1,406; while the limits of accommodation are given at 1,144, so that there must have been considerable overcrowding. There were 227 first admissions; 61 readmissions; 116 recoveries; 27 discharged relieved; and 95 deaths. The recoveries stand at 40·3 per cent. on the admissions, and the deaths at 6·9 per cent. on the average number resident. Only 15 of the deaths were caused by cerebral and spinal diseases, but 44 were due to consumption and 10 to pneumonia. Various moral causes account for 42 of the year's admissions; intemperance in drink for 40; hereditary influence for 71; and congenital defect for 10. Dr. Woods admits that the death-rate from consumption is too high, but he does not say what steps are being taken to prevent or combat the effects of overcrowding. No doubt the open air treatment would be extremely difficult or practically impossible to carry out in its entirety in asylums, but surely something could be done in that direction. As new buildings to accommodate 285 patients are about to be erected, it is to be hoped that this comparatively new idea of the treatment of phthisis will not be altogether lost sight of. It is suggested that some method of warming and ventilation be adopted in the present building; but it has not yet been decided which of the ordinary methods to adopt. No doubt in large wards some "system" is desirable, but nothing can take the place of open fireplaces. Dr. Woods has four assistant medical officers, one of whom is a lady. The cost of maintenance per patient per annum was £21 8s. 2d.

